# I don’t know what type of arthritis I have: A population-based comparison of people with arthritis who knew their specific type and those who didn’t

**DOI:** 10.1371/journal.pone.0270029

**Published:** 2022-06-21

**Authors:** Elizabeth M. Badley, Jessica M. Wilfong, Christina H. Chan, Mayilee Canizares, Anthony V. Perruccio

**Affiliations:** 1 Arthritis Community Research and Evaluation Unit, Krembil Research Institute, University Health Network, Toronto, Canada; 2 Schroeder Arthritis Institute, Krembil Research Institute, University Health Network, Toronto, Canada; 3 Dalla Lana School of Public Health, University of Toronto, Toronto, Canada; Emory University School of Medicine, UNITED STATES

## Abstract

**Objective:**

To understand differences between people with arthritis who do not know their type (DK) compared to those reporting osteoarthritis (OA) or inflammatory and autoimmune types of arthritis (IAA), including the receipt of appropriate health care, information, and services.

**Methods:**

Analysis of the Survey on Living with Chronic Disease in Canada–Arthritis Component. Respondents aged ≥20 years with health professional-diagnosed arthritis (n = 4,385) were characterized as reporting DK, OA or IAA. Variables: arthritis characteristics (duration, number and site of joints affected), arthritis impact (current pain and fatigue, difficulty in sleeping and daily activities, impact on life), health (self-rated general and mental health, life stress), arthritis management strategies (seeing health professionals, medication use, assistive devices, receipt of arthritis information, self-management activities). Multinomial logistic and log-Poisson regressions were used, as appropriate, to compare the DK to the OA and IAA groups.

**Results:**

In this arthritis sample, 44.2% were in the DK group, 38.3% reported OA and 17.5% reported IAA. Those in the DK group were more likely to be younger, have low income, low education, and be of non-white cultural background compared to those with OA. There were no significant differences in arthritis impact, but the DK group was less likely to have received information on, or have used, arthritis management strategies.

**Conclusions:**

The sociodemographic characteristics of the DK group suggest they likely have lower health literacy. They were less likely to have accessed health care and other support services, indicating this is an important group for health education, both for individuals with arthritis and health care providers.

## Introduction

Population-based studies consistently show arthritis to be one of the most frequently reported chronic health conditions and a major cause of disability [1]. In Canada, as elsewhere, the prevalence is higher in women, those with lower socio-economic status, and increases with age [2–5]. The prevalence of arthritis is likely to rise with an aging population [6]. Arthritis is characterized by pain, swelling and stiffness of the joint. The arthritis family consists of more than one hundred specific conditions. The two most ‘important’ categories of arthritis, albeit for different reasons, are inflammatory and autoimmune types of arthritis (IAA) and osteoarthritis (OA). IAAs are important as they represent a group of serious medical conditions which affect other systems of the body as well as the joints. The various types of IAA are usually managed by specialists, most commonly rheumatologists, using a range of disease modifying drugs that need close monitoring. Rheumatoid arthritis (RA) is the hallmark form of inflammatory arthritis, affecting about 0.5% of the population [7]. Taken together the overall population prevalence for the total IAA category is likely to be, at most, about 2% [1, 4, 8]. OA is important as it is by far the most common type of arthritis and is estimated to affect more than 1 in 8 of the adult population in Western countries [1, 9, 10]. OA is characterized by degeneration of the cartilage in the joints, leading to pain, deformity, and limitation of activity. To date there are no specific medical therapies for this condition, other than non-steroidal anti-inflammatory drugs [11, 12]. Joint replacement surgery is only indicated for those with severe end-stage OA [13]. Because of its high frequency, it is the most common cause of physical disability in the population, with high costs particularly associated with the need for total joint replacement surgery [1, 3, 14]. It is estimated to account for about 70% of all arthritis diagnoses [15].

Information about the size and nature of the impact of arthritis in the population is crucial to ensuring adequate and appropriate services to meet the needs of those affected, especially for the delivery and implementation of population-based interventions. Population-based estimates for individual types of arthritis are difficult to obtain. Health services administrative data on physician visits, which rely on information from routine clinical consultations, have only limited demographic data with little or no other information on the life situation of the patients or the impact of the disease. Representative information about the distribution of risk factors, and prevalence and impact of health conditions is therefore generally acquired through population surveys. However, there is some concern with questions about specific types of arthritis, as a sizeable proportion of people with arthritis report that they do not know the type of arthritis [16]. A study of prevalence trends for arthritis in the US from 1999–2014 gave an age-adjusted prevalence of arthritis of 24.7%, with a prevalence of only 9.7% for self-reported OA and 8.0% for ‘don’t know’ type of arthritis [17]. Similarly, a study from Australia showed an overall prevalence for arthritis of 21.6%, with 11.7% of the population reporting OA and 6.5% not knowing their type [18].

The characteristics of people with arthritis who do not know their type has been little studied. Knowing one’s type of arthritis is important for accessing appropriate care and advice and managing the symptoms of the disease. Activities related to self-management are important for people with arthritis as the mainstays of care for all types of arthritis and include pain control, engaging in physical activity and maintaining a healthy weight [13, 19]. Furthermore, individuals who do not know their type of arthritis are more likely to have inadequate functional health literacy compared to the general population [20]. Having lower functional health literacy is associated with difficulty in managing chronic illness, and in particular access to self-management-based interventions [21, 22].

This study takes advantage of an arthritis-focused supplement of the Canadian Community Health Survey, a representative national health survey, which asked individuals with self-reported doctor-diagnosed arthritis about the type of arthritis, including not known type. The objective of this present study is to understand differences between people with arthritis self-reporting knowing and not knowing the specific arthritis diagnosis, including the receipt of appropriate health care, information, and services.

## Methods

### Data sources

Data for analyses were obtained from the 2009 Survey on Living with Chronic Diseases in Canada–Arthritis Component (SLCDC-A) [23]. This is a cross-sectional survey that was conducted as an extension of the 2008 Canadian Community Health Survey (CCHS) to document the experiences of Canadians living with arthritis [24]. The CCHS is an annual cross-sectional survey, which uses a complex cluster design to generate a nationally representative sample of the household population with an estimated coverage of approximately 98%. The sample for the Arthritis Component of the 2009 SLCDC was drawn from respondents aged 20 years and older living in one of Canada’s ten provinces answering affirmatively to an arthritis question in the CCHS, “Do you have arthritis, excluding fibromyalgia?”. This was asked as part of a series of questions about long-term health conditions diagnosed by a health professional that had lasted or were expected to last for 6 months or longer. Details about the sampling methods of the SLCDC-A are documented elsewhere [25]. The questions for the SLCDC-A were developed by the Public Health Agency of Canada and Statistics Canada in consultation with an expert working group of clinicians and researchers. The survey was administered by trained personnel via a structured telephone interview in February and March 2009. Respondents were initially offered to complete the interview in either English or French. To remove language as a barrier to conducting interviews, the regional offices recruited interviewers with a wide range of language competencies and when necessary, cases were transferred to an interviewer with the language competency needed to complete an interview. Data from the SLCDC-A were also linked to the respondents’ data in the CCHS. A total of 4,565 respondents with arthritis consented to participate and share their linked data with partnering organizations (Public Health Agency of Canada, Health Canada and provincial governments): 78.4% participation rate.

This study is based on analyses of previously de-identified data collected by Statistics Canada and accessed through their Research Data Centre (Toronto). RDCs are operated under the provisions of the Statistics Act in accordance with all the confidentiality rules. The data were made available for this study through a formally reviewed research proposal to Statistics Canada, and in view of this the University Health Network Research Ethics Board waived the requirement for institutional ethics approval.

### Identification of type of arthritis

Respondents to the SLCDC-A were asked whether they knew what kind of arthritis they had, with an initial question with the response options yes or no. Only those who responded yes were then asked to specify the type with the following response options: osteoarthritis, rheumatoid arthritis, ankylosing spondylitis, gout, lupus, polymyalgia rheumatica, polymyositis, psoriatic arthritis, Reiter’s syndrome, scleroderma/systemic sclerosis, Sjogren’s syndrome, vasculitis, fibromyalgia, bursitis/carpal tunnel/tendonitis, and other. We categorized individuals as not knowing their type of arthritis (DK) (unweighted n = 1925), having OA only (OA) (unweighted n = 1749), having an inflammatory and autoimmune type of arthritis (IAA) (rheumatoid arthritis, ankylosing spondylitis, lupus, polymyalgia rheumatica, polymyositis, psoriatic arthritis, Reiter’s syndrome, scleroderma/systemic sclerosis, Sjogren’s syndrome, vasculitis) (unweighted n = 711) and having another type of arthritis (gout, fibromyalgia, bursitis/carpal tunnel/tendonitis, and ‘other’ (unweighted n = 180). The latter group was excluded from analyses due to the small sample size and the miscellany of conditions included.

### Sociodemographic and health behaviour variables

Data on sociodemographic and health behaviour characteristics were incorporated from the 2008 parent CCHS. Information on the survey questions and analysis groups used for each sociodemographic and health behaviour variable are given in S1 Table.

#### Sociodemographic

Highest level of education was dichotomized into secondary school or less and at least some post-secondary. Household income was provided by Statistics Canada by decile (i.e. ten categories including approximately the same percentage of residents for each province) based on the ratio of their total household income to the low income cut-off corresponding to their household and community size. For analyses, the three lower deciles were used to classify people in the low income category. Marital status was categorized as married/common-law, widowed/separated/divorced, and single, never married. Cultural background was derived from a question which asked respondents to identify their cultural and racial background from a list of 13 groups. Three mutually exclusive groups were derived: only white, only Aboriginal (North American Indian, Metis, Inuit), and other (Black, Korean, Filipino, Japanese, Chinese, South Asian, Southeast Asian, Arab, West Asian, Latin American, other, and those of multicultural origin). In our sample the largest non-white group is the Asian population (11.9%) with only a very small proportion who identify as Black (2.3%) or Latin American (1.2%)), consistent with what is found in the Canadian census [26]. Urban residence was determined by whether a respondent lived in an area that had a population concentration of 1000 or more and a density of 400 people per square kilometer.

#### Health behaviours

Self-reported height and weight were used to calculate Body Mass Index (BMI = weight(kg)/height(m)^2^). BMI was categorized as underweight/normal (<25 kg/m^2^), overweight (25–29.9 kg/m^2^) and obese (≥30 kg/m^2^) [27]. Physical activity was classified as active, moderately active, or inactive, using Statistics Canada’s Physical Activity Index derived from total daily energy expenditure values calculated for 21 leisure time activities [28]. Smoking status (current/former smoker, never smoked) was determined based on reported lifetime cigarette consumption. Alcohol consumption (regular drinker, non-regular drinker) was determined based on reported frequency of drinking alcoholic beverages in the past 12 months.

### Arthritis-related variables

Data on arthritis-related variables were incorporated from the SLCDC-A. Information on the survey questions and analysis groups used for each arthritis-related characteristic are given in S2 Table.

Duration of arthritis was calculated as the difference between the age at which respondents reported they were diagnosed with arthritis and their age at the time of the survey, and was grouped as 0–5, 6–10, 11–19, and 20+ years. Respondents were asked to report if they had experienced joint pain in the past month, and if so, to indicate which joints have been painful from a list of 19 joints: right and left shoulder, elbow, wrist, hand/fingers/thumb, hip, knee, ankle, foot/toes, and neck, back, and other. Individual joints were grouped into sites (i.e. one or both knees) for a total of 11 sites including the neck and back. A count of painful joint sites was calculated and was grouped into 0, 1, 2–3, and 4+ sites. Additionally, specific limb joint pain was coded as symmetrical (both left and right) or single (only left or right).

#### Impact of arthritis

Respondents were asked “In the past month, how often have you experienced joint pain?” with response options “always”, “often”, “sometimes”, “rarely” or “never”. Those who reported experiencing joint pain were then asked to rate the average level of pain they experienced over the past month on a scale of 1 to 10, with 1 meaning “little pain” and 10 meaning “pain as bad as it could be”. For analytic purposes, severe joint pain was defined as a pain intensity of 7 or more out of 10 and frequent if experienced ‘always’ or ‘often’ [29]. The intensity and frequency of fatigue experienced was assessed in a similar manner, with the same cut-offs for severe and frequent fatigue. A question was also asked about limitations in getting a good night’s sleep due to arthritis in the past month as “a lot”, “a little” or “not at all” and was dichotomized to indicate sleep was affected a lot or sleep was not affected a lot. Limitations in instrumental activities of daily living were assessed by asking about limitations due to their arthritis in five activities with response options of limited “a lot”, “a little” and “not at all”. Severe limitations were defined as being limited “a lot” in three or more of these daily living activities [29]. Respondents rated their own health and mental health as “excellent”, “very good”, “good”, “fair”, or “poor”. Suboptimal health was defined as having “fair” or “poor” general or mental health [29]. Respondents were also asked to indicate how much their arthritis impacts their life overall with response options “not at all”, “a little bit”, “moderately”, “quite a bit”, or “extremely”. Those who responded with the latter two categories were considered to be impacted greatly. Respondents were also asked to indicate the amount of stress in their lives with response options “not at all stressful”, “not very stressful”, “a bit stressful”, “quite a bit stressful”, and “extremely stressful”. The latter two categories were combined to indicate high life stress.

#### Health care and other self-management strategies

Details of the questions on healthcare, medication and assistive device use, receipt of information and self-management variables are provided in S3 Table.

*Health care utilization*. Respondents were asked if they had seen or talked to a 1) family doctor or general practitioner (referred to primary care physicians throughout to reflect current preferred terminology); 2) orthopaedic surgeon; 3) rheumatologist; 4) internist; 5) physiotherapist or occupational therapist; 6) pharmacist; and 7) complementary practitioner in the past 12 months for their arthritis (response options: yes/no).

*Medication use*. Respondents were asked if they took 1) prescription medications; 2) non-prescription medications; and 3) natural health products in the past month for their arthritis (response options: yes/no).

*Assistive device use*. Respondents were asked if they currently used assistive devices to help with usual activities. The types of assistive devices included were devices for walking or getting around, devices for dressing, orthotics, a built up or special tool (such as a jar opener or a reacher), a built up or special chair, a safety device (such as bathtub grab bar or hand rail), or any other. A variable was derived by Statistics Canada to indicate the use of any assistive device for arthritis.

*Information received*. Respondents were provided the following prompt: “The next few questions are about information you may have received to help you manage your arthritis. (By manage, we mean things that may help you cope with your arthritis, improve any arthritis symptoms you may have, or keep further problems from developing.)” Questions were then asked about information received about 1) the type of arthritis they have; 2) how to manage their arthritis; 3) the emotional impact of arthritis; 4) where to receive support for coping with arthritis; and 5) if they felt they had received enough information to manage their arthritis (response options: yes/no).

*Arthritis self-management*. Respondents were asked if 1) they had ever taken a course or class on how to manage arthritis; 2) whether they currently exercise or participate in physical activity; 3) whether they are currently trying to control or lose weight; and 4) whether they have used community-based facilities and programs in the past 12 months (response options: yes/no).

### Analysis

Descriptive analyses were performed to obtain distributions of study variables among the arthritis groups and were weighted using survey weights generated by Statistics Canada to be representative of the Canadian population aged 20+ with arthritis [24]. Bootstrap weights provided by Statistics Canada were used to estimate statistical significance taking into account the complex sampling design. The bootstrap re-sampling technique is commonly used to obtain appropriate variance estimates in the presence of potential clustering, stratification, and unequal selection probabilities [30, 31]. We used a multinomial logistic regression to compare select sociodemographic, lifestyle, and arthritis-related characteristics between the types of arthritis groups. We used multivariable adjusted log-Poisson regressions to compare the prevalence of various outcomes (as prevalence ratios) between arthritis groups controlling for age, sex, education, income, marital status, cultural background, area of residence, BMI, smoking, physical activity, alcohol consumption, duration of arthritis, and number of joint sites. The data was prepared using SAS version 9.4 and data analyses were carried out using STATA 16.

## Results

Almost half (44.2%) of the arthritis sample reported not knowing their type of arthritis, 38.3% reported having OA and 17.5% reported having IAA.

Table 1 presents the characteristics of the DK, OA, and IAA groups. The DK and IAA groups had a similar age distribution but compared to those with OA the distribution for the DK group was skewed to younger ages. The DK group had higher proportions of respondents who were males, individuals with lower education levels and lower household income, and a higher proportion of non-white people, than those in the OA group. There were no significant differences between the DK and IAA groups.

**Table 1:** Distribution of sociodemographic and health behaviours of respondents with arthritis, Survey on Living with Chronic Diseases in Canada–Arthritis Component, 2009.

	Type of arthritisa				
	Estimated Percent (%)b			P-value	
Characteristic	DK	OA	IAA	OA vs DK	IAA vs DK
	n = 1,520,500^b^	n = 1,318,000^b^	n = 602,500^b^		
**Sociodemographic**					
Age					
20–44	13.7	7.2	14.0	**0.021**	0.111
45–54	16.3	19.9	24.3		
55–64	26.1	28.3	25.9		
65–74	23.2	25.1	20.8		
75+	20.7	19.5	15.0		
Sex					
Male	43.6	28.1	36.3	**<0.001**	0.069
Female	56.4	71.9	63.7		
Education					
Secondary or less	44.7	35.8	39.9	**0.005**	0.268
At least some post-secondary	55.3	64.2	60.1		
Low household income					
Yes	34.5	27.9	35.9	**0.003**	0.648
No	50.3	61.3	51.9		
Not reported	15.1	10.8	12.2		
Marital status					
Married/Common law	68.0	67.8	67.9	0.931	0.439
Widowed/Separated/Divorced	24.3	25.0	21.8		
Single, never married	7.7	7.2	10.3		
Cultural background					
White	80.8	93.0	88.1	**<0.001**	0.161
Aboriginal	4.6	2.6	3.6		
Other	14.6	4.4	8.3		
Area of residence					
Rural	20.7	22.5	21.5	0.398	0.779
Urban	79.3	77.5	78.5		
**Health behaviours**					
BMI					
Underweight/Normal	29.8	33.8	38.0	0.350	0.123
Overweight	40.9	39.6	37.8		
Obese	29.3	26.6	24.2		
Smoking status					
Current/Former	67.9	68.7	72.1	0.801	0.316
Physical activity					
Inactive	57.7	54.1	57.9	0.261	0.952
Alcohol					
Regular drinker	50.7	56.9	52.0	**0.049**	0.764
**Arthritis-related**					
Age at diagnosis					
< 45 years old	36.6	30.5	49.3	**0.004**	**0.004**
45–54 years old	20.5	30.2	22.0		
55–64 years old	23.3	23.1	16.3		
65+ years old	19.6	16.3	12.4		
Duration of arthritis					
0–5 years	40.0	34.3	29.6	0.272	**0.006**
6–10 years	22.0	22.0	20.6		
11–19 years	20.8	24.0	20.7		
20+ years	17.1	19.8	29.1		
Number of painful joint sites					
0	11.6	5.8	12.1	**<0.001**	**0.012**
1	19.4	15.2	10.4		
2–3	35.1	36.6	32.8		
4+	33.9	42.3	44.8		

^a^DK = don’t know type; OA = osteoarthritis; IAA = inflammatory and autoimmune types of arthritis.

^b^Weighted estimate.

^c^Significance of chi-square tests (indicated in bold (p<0.05)) based on weighted data.

**Fig 1:**
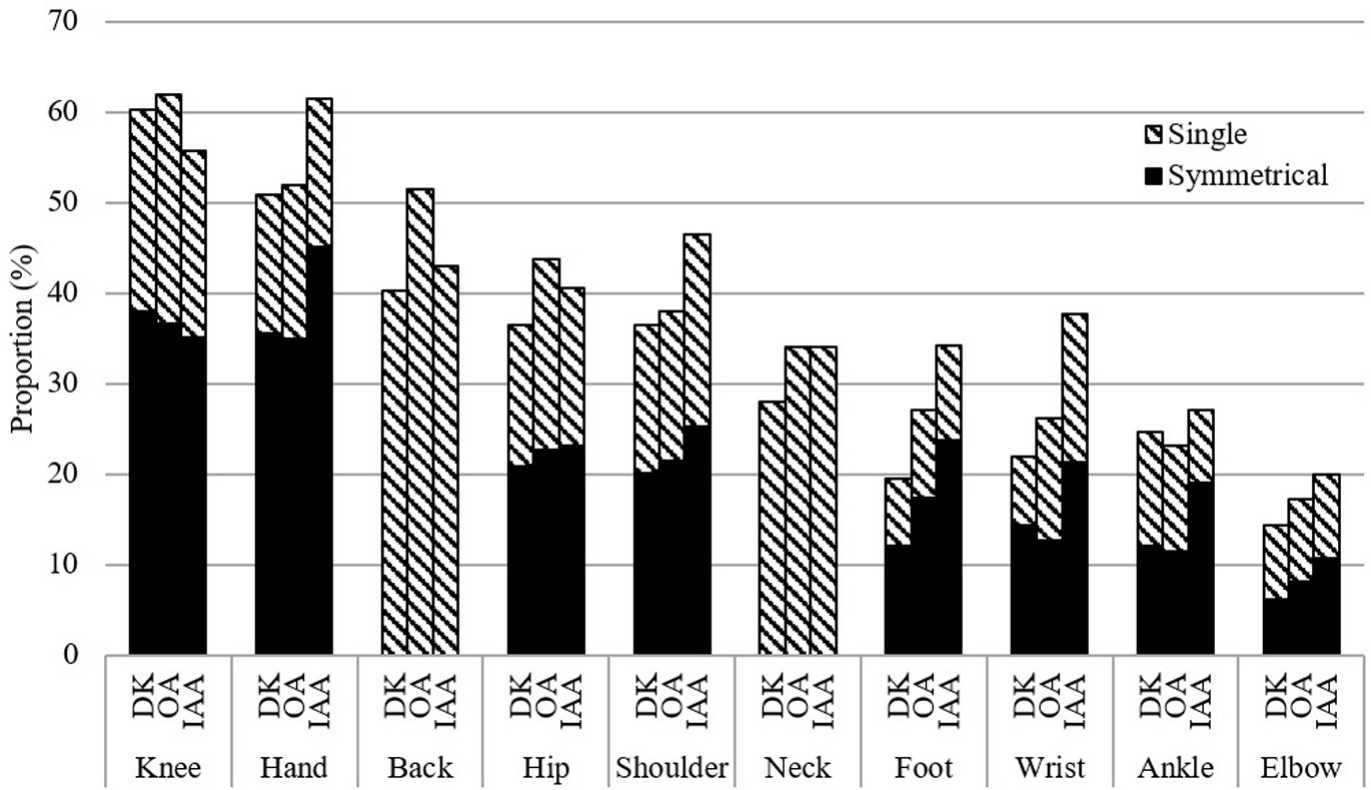
Pattern of painful joint sites for specific arthritis types^a^, Survey on Living with Chronic Diseases in Canada–Arthritis Component, 2009. ^a^DK = don’t know type; OA = osteoarthritis; IAA = inflammatory and autoimmune types of arthritis.

The mean age at diagnosis of the DK group (M = 50.1, SD = 15.7) was somewhat older than that of the IAA group (M = 44.0, SD = 18.8) (p<0.001), but was not significantly different for that for OA (M = 50.4, SD = 13.9) (p = 0.524). However, the distribution of age at diagnosis differed somewhat between the DK and OA groups (Table 1). Overall, the mean duration of disease was shorter for the DK group, (M = 11.0, SD = 10.6) compared to the OA group (M = 12.0, SD = 10.5) (p = 0.002) and the IAA group (M = 15.2, SD = 14.2) (p<0.001). The DK group had higher proportions with duration of arthritis in the 0–5 year range. The proportion of the DK group reporting only one symptomatic joint site was significantly higher than the OA or IAA groups. The mean total number of joint sites affected for the DK group (M = 3.0, SD = 2.4) was significantly lower compared to those with OA (M = 3.5, SD = 2.4) (p<0.001) and those with IAA (M = 3.6, SD = 2.7) (p<0.001). Nevertheless, the distribution of painful joint sites was similar particularly between the DK and OA groups (Fig 1). For both groups the most frequently affected painful joint sites were the knee, hand, and back. The elbow was the least frequently reported joint. The IAA group had somewhat higher proportions for the hand, shoulder, foot and wrist, consistent with the expected pattern of joint involvement for RA [32], the most frequently reported diagnosis in this group.

Bringing the above data together, Table 2 provides odds ratios for the probability of OA and IAA vs DK. Compared to the DK group those with OA were significantly less likely to be in the youngest age group (20–44), to be male, to have secondary or less education, to have low household income or to not report their household income and to have non-white cultural background, with no difference in duration of arthritis. Comparison of the IAA and DK groups showed those with IAA were more likely to be aged 45–55 years and have a duration of arthritis of 20 or more years. Both the OA and IAA groups were less likely than the DK group to have a lower number of painful joints.

**Table 2:** Multinomial logistic regression comparing those with OA and IAA to those in the DK group^a^, Survey on Living with Chronic Diseases in Canada–Arthritis Component, 2009.

	OA vs. DK		IAA vs. DK	
	OR	95% CI	OR	95% CI
Age (ref: 75+ years years)				
20–44 years	**0.52**	**0.33, 0.84**	1.80	0.95, 3.44
45–54 years	1.26	0.76, 2.09	**2.66**	**1.35, 5.24**
55–64 years	1.02	0.69, 1.49	1.53	0.82, 2.86
65–74 years	1.14	0.84, 1.55	1.34	0.78, 2.33
Sex (ref: Female)				
Male	**0.45**	**0.35, 0.59**	0.74	0.53, 1.05
Education (ref: At least some post-secondary)				
Secondary or less	**0.65**	**0.48, 0.88**	0.83	0.57, 1.23
Low household income (ref: No)				
Yes	**0.65**	**0.48, 0.89**	1.00	0.64, 1.57
Not reported	**0.51**	**0.34, 0.77**	0.80	0.44, 1.45
Cultural background (ref: White)				
Aboriginal	0.49	0.23, 1.06	0.54	0.24, 1.19
Other	**0.21**	**0.08, 0.59**	0.45	0.16, 1.24
Duration of arthritis (ref: 20+ years)				
0–5 years	0.98	0.71, 1.34	**0.44**	**0.27, 0.71**
6–10 years	0.90	0.67, 1.22	**0.50**	**0.31, 0.81**
11–19 years	1.18	0.80, 1.74	**0.59**	**0.35, 1.00**
Number of painful joint sites (ref: 4+ sites)				
0 joints	**0.51**	**0.32, 0.81**	1.08	0.57, 2.04
1 joint	**0.64**	**0.46, 0.90**	**0.43**	**0.26, 0.71**
2–3 joints	0.91	0.68, 1.23	0.81	0.55, 1.21

^a^DK = don’t know type; OA = osteoarthritis; IAA = inflammatory and autoimmune types of arthritis Significant (p<0.05) odds ratios (OR) indicated by bold typeface.

There were no significant differences between the DK, OA, and IAA groups in any of the indicators of arthritis impact (Table 3). These indicators were the proportions with severe and frequent pain or fatigue, reporting a lot of sleep limitations and severe activity limitations. There were also no significant differences between the overall health indicators of self-rated general health, self-rated mental health, overall impact of arthritis, and life stress between the groups.

**Table 3:** Impact of arthritis among specific arthritis types^a^, Survey on Living with Chronic Diseases in Canada–Arthritis Component, 2009.

	DK	OA	IAA	OA vs DK	IAA vs DK
Outcome	%	%	%	PR (95% CI)^b^	PR (95% CI)^b^
Joint pain					
Severe and frequent	27.1	26.3	30.3	0.97 (0.78, 1.20)	0.99 (0.75, 1.30)
Fatigue					
Severe and frequent	18.4	19.3	24.4	1.01 (0.76, 1.33)	1.09 (0.81, 1.45)
Sleep					
Affected a lot	23.0	25.3	26.8	1.12 (0.88, 1.42)	0.96 (0.73, 1.25)
Activities					
≥3 activities limited a lot	8.9	12.6	11.7	1.25 (0.87, 1.80)	1.12 (0.76, 1.64)
Self-rated general health					
Fair/poor	30.8	27.8	35.8	1.02 (0.84, 1.23)	1.12 (0.88, 1.43)
Self-rated mental health					
Fair/poor	14.0	10.7	9.1	0.92 (0.59, 1.44)	0.63 (0.38, 1.03)
Overall impact of arthritis					
Quite a bit/extremely	21.8	24.0	31.1	1.19 (0.92, 1.54)	1.25 (0.95, 1.63)
Life stress					
Quite a bit/extremely	19.4	19.3	25.3	0.83 (0.66, 1.04)	0.96 (0.73, 1.26)

^a^DK = don’t know type; OA = osteoarthritis; IAA = inflammatory and autoimmune types of arthritis

^b^Adjusted for age, sex, education, income, marital status, cultural background, area of residence, BMI, smoking, physical activity, alcohol consumption, duration of arthritis, and number of joint sites with DK as the reference group.

The major differences between the groups were seen in health care use, medication, and other types of arthritis management (Table 4). Compared to the DK group, those with OA were more likely to have seen an orthopedic surgeon, and those with OA or IAA more likely to have seen a rheumatologist for their arthritis in the past year, particularly the IAA group. As might be expected given the type of treatment, those with IAA were more likely to be taking prescription medication. They were also more likely to be using any type of assistive device. Those knowing their type of arthritis were more likely to report that they had received information about their type of arthritis, arthritis management, emotional impact of arthritis and where to find support for their arthritis. There were no significant differences in the proportion of individuals feeling that they had enough information. In terms of arthritis self-management, individuals reporting knowing their type of arthritis were more likely to have taken classes, to exercise (IAA), to be trying to lose or control their weight (OA) or use other facilities, services or programs.

**Table 4:** Health care, medication and assistive devices use, types of information received, and self-management for arthritis reported by Canadians with specific arthritis types^a^, Survey on Living with Chronic Diseases in Canada–Arthritis Component, 2009.

	DK	OA	IAA	OA vs DK	IAA vs DK
Outcome	%	%	%	PR (95% CI)^b^	PR (95% CI)^b^
**Health care use in past year for arthritis**					
Primary care physician	63.0	66.9	69.4	1.02 (0.93, 1.11)	1.07 (0.96, 1.19)
Orthopaedic surgeon	10.2	17.1	15.9	**1.64 (1.20, 2.24)**	1.37 (0.93, 2.01)
Rheumatologist	3.7	7.4	24.5	**1.77 (1.02, 3.06)**	**5.80 (3.30, 10.18)**
Internist	3.8	4.1	4.1	1.08 (0.58, 1.99)	1.03 (0.52, 2.04)
Physio or occupational therapist	13.2	22.1	20.1	**1.44 (1.08, 1.93)**	1.28 (0.91, 1.81)
Pharmacist	22.5	20.0	27.2	0.86 (0.67, 1.11)	1.11 (0.84, 1.46)
Complementary practitioners	11.9	16.3	15.2	1.04 (0.76, 1.41)	0.96 (0.65, 1.41)
**Medication for arthritis**					
Prescription	31.6	39.4	52.6	1.16 (0.99, 1.36)	**1.55 (1.30, 1.83)**
Non-prescription	60.3	66.2	63.4	1.04 (0.96, 1.14)	1.03 (0.91, 1.18)
Natural product	36.7	44.8	36.6	1.13 (0.97, 1.32)	0.93 (0.77, 1.13)
**Assistive devices for arthritis**					
Any	37.0	48.7	49.6	1.14 (1.00, 1.30)	**1.21 (1.01, 1.44)**
**Receiving info on**					
Type of arthritis	24.3	57.3	63.0	**2.32 (1.85, 2.90)**	**2.47 (1.95, 3.14)**
Arthritis management	54.1	67.5	76.3	**1.18 (1.07, 1.31)**	**1.36 (1.21, 1.53)**
Emotional impact	8.7	13.9	19.9	1.35 (1.00, 1.82)	**2.03 (1.44, 2.84)**
Where to find arthritis support info	12.0	18.2	24.0	**1.44 (1.07, 1.93)**	**1.92 (1.34, 2.74)**
Feel has enough arthritis info	82.3	82.4	80.2	0.98 (0.92, 1.05)	0.98 (0.90, 1.06)
**Arthritis self-management**					
Taken class	4.2	12.1	14.1	**2.20 (1.42, 3.42)**	**2.57 (1.56, 4.24)**
Exercise	58.0	65.8	69.5	1.10 (0.99, 1.21)	**1.19 (1.05, 1.34)**
Lose/control weight	53.1	58.1	56.1	**1.13 (1.03, 1.23)**	1.14 (0.99, 1.32)
Use facilities/services/programs	7.9	15.0	13.4	**1.44 (1.05, 1.97)**	1.41 (0.98, 2.01)

^a^DK = don’t know type; OA = osteoarthritis; IAA = inflammatory and autoimmune types of arthritis

^b^Adjusted for age, sex, education, income, marital status, cultural background, area of residence, BMI, smoking, physical activity, alcohol consumption, duration of arthritis, and number of joint sites with DK as the reference group.

Significant (p<0.05) prevalence ratios (PR) indicated by bold typeface.

## Discussion

When asked directly, almost half of respondents in a representative sample of people living with arthritis in Canada said they did not know the specific type of arthritis they were diagnosed with. This raises questions about the differences between those who knew and the large proportion of those who did not know their arthritis diagnosis and the consequences for the management of their arthritis.

Individuals with arthritis not knowing their specific diagnosis differed somewhat in their personal characteristics from those with OA, although were similar to the much smaller group with IAA. They were more likely to be male, be younger than 45 years, have lower levels of education, lower income, and report non-white cultural backgrounds compared to those with OA. Similar findings with regard to differences by demographic status were also found in a study of general health literacy in the Australian population [33], and in a study of patients with musculoskeletal pain [22]. A study of health literacy in orthopedic patients also found lower scores for health literacy in those with less than college education, non-Caucasians, and females but no difference by age [34]. Low health literacy has been shown to be associated with a decreased likelihood of recalling health information, such as a diagnosis [35]. This is consistent with low health literacy contributing to not knowing a specific arthritis diagnosis. It is likely that those with lower health literacy would have fewer resources to proactively seek information to help them manage their disease.

While similar demographic characteristics, particularly age distribution, were found for the DK and IAA groups, this is likely for different reasons. The relatively higher proportion of younger individuals and the younger mean age at diagnosis in the IAA group is compatible with the expected mean age of onset of RA and other conditions in this group [7, 36]. The IAA group also represents serious health conditions requiring treatment that need regular monitoring by specialists, increasing the probability that patients know their diagnosis. Individuals in the DK group, while younger, had a similar age at diagnosis to the OA groups. Being younger may affect the provision of information by health care providers, particularly in view of a common perception of arthritis, particularly OA, as a disease of the elderly [37]. A qualitative study of younger (age 35–65 years) individuals with OA indicated that particularly those aged 35–49 years were frustrated by the lack of health care opinions and advice [38]. They felt a health care-system focused on late-stage disease offered little guidance, leaving them to seek solutions on their own account.

In Canada, primary care physicians are the gatekeepers to medical interventions, including referral to specialists, as would be necessary for those with IAA. Therefore, for most conditions, a preliminary diagnosis is made by primary care physicians. Referrals to specialists are only made for those needing complex interventions such as treatment with biologic and other medications by rheumatologists, or orthopedic surgeons for total joint replacement surgery. Representative data from the population shows that over 85% of the Canadian population have a regular healthcare provider, usually a primary care physician [39]. In this study over 60% reported seeing a primary care physician in the prior year specifically for their arthritis. A study of physician billing in Ontario, Canada showed the majority, 78%, of arthritis billings were in primary care. Looking at specific types of arthritis, most (79%) billings for OA were by primary care physicians. The proportion for IAA conditions was lower with a higher percentage of billings by specialists [40]. Data from billing records in primary care also show a high use of non-specific arthritis codes [40]. The education of primary care physicians in the diagnosis and management of arthritis has been found to be lacking, and doctors lack confidence in the diagnosis and management of OA and RA [41, 42]. Studies from the UK show that diagnoses of OA of the knee, hip and hand are under-represented in primary care clinical records [43–46]. It is possible that communication of non-specific arthritis diagnoses to patients could contribute to the lack of knowing their specific diagnosis. Additionally, given that almost a quarter of respondents who did not know their arthritis type reported yes to a question about whether they had received information on their type of arthritis, it may be that they recall being told about the type and being provided with information, but did not recall the name at the time of the survey.

Logistic regression findings showed no difference in arthritis impact in terms of pain, fatigue, limitations in activity and impact on quality of life between those in the DK, OA, and IAA groups. Nevertheless, not knowing the type of arthritis had consequences for those in the DK group. People who did not know their type of arthritis were less likely to have accessed health care and other support services in the past year. While a similar proportion had seen their primary care physician, they were less likely to have seen a specialist. People who did not know their type of arthritis were less likely to have accessed other support services. They were less likely to have received information about their arthritis, arthritis management and support, and less likely to have taken classes, exercise, or access support services and programs for arthritis. It may be that not knowing the specific diagnosis could be a barrier to accessing care, services, and resources for arthritis management. Conversely, while all respondents must have accessed services to receive their diagnosis of arthritis, it may be that ongoing barriers to care prevented reinforcement or clarification on the type.

Clearly, it is not possible to know what kind of arthritis the DK group was likely to have. While the DK group may include individuals with IAA, we think this is likely to be only a minority as, as noted in the introduction, the population prevalence of IAA is low. Also, most types of IAA are treated with drugs that have significant side effects that require frequent clinical monitoring, so these individuals likely know their type of arthritis. OA is the predominant type of arthritis in the population, affecting about 70% of those with arthritis [15]. Therefore, considering the much higher prevalence of OA, we suggest that, on balance, the DK group is likely to be comprised mostly of individuals with OA. Additional circumstantial evidence that people not knowing their type of arthritis might have OA comes from a study of time trends in the prevalence of various types of arthritis in the United States [17]. In this study, Park et al. described a recent (2010–2015) increase in prevalence of OA, which was mirrored by a decrease in the proportion not knowing their diagnosis. This decrease was significant for women, people with low income and low education and obese people. This was also in a period where there appears to be growing medical interest in OA, as reflected by an increased proportion of scientific papers related to this condition [47].

The major strength of this study is that it used data from a nationally representative survey of individuals who currently have arthritis and clearly separates those who do not know the kind of arthritis they have from those that do. As far as we can tell this is the first study which has compared the arthritis-related characteristics of those who do and do not know their specific arthritis diagnosis. An inevitable limitation is that this study is a cross-sectional population-based survey so that it is impossible to determine the directionality of any associations. In addition, disease diagnosis was self-reported without clinical confirmation. Though self-reported type of arthritis may result in misclassification at an individual level, validation studies have shown reasonable reliability of self-reported arthritis as a whole [48]. A review of the effect of OA definition on prevalence showed similar estimates for clinical symptomatic and self-reported OA [49], with reasonable reliability for self-report [50]. The population prevalence of self -reported IAA (2.9%) was higher than expected from epidemiologic estimates. Other studies have found RA in particular to be over-reported in population health surveys [17]. The proportion reporting not knowing their type of arthritis (44.2%) in the present study was slightly higher than in the studies of Gill et al. and Park et al. (approximately 30% in both studies) [17, 18]. This could be a result of the sequence of questioning. In the SLCDC-A respondents were first asked whether they knew their type of arthritis, and only those responding affirmatively were asked about the type, so there was no initial prompting using specific arthritis diagnoses. The prevalence of arthritis tends to be higher in studies where respondents are simply presented with a list of types of arthritis rather than asking them if they know their type first. For example, in Canada, a change of wording of the arthritis question used in national population health surveys from asking about whether a respondent had health professional diagnosed arthritis, to one which gave examples of types of arthritis, resulted in an increase in the estimated prevalence of arthritis from about 17% to 20% [51].

From the perspective of understanding the impact of different kinds of arthritis in the population, relying exclusively on self-report of specific diagnoses and neglecting those who do not know the type may lead to an underestimate of the total burden, particularly for OA. Understanding the full population impact of OA is important for the development of appropriate education and care interventions and setting of research priorities. Despite a considerable impact in terms of pain, disability and fatigue, individuals who did not know their type of arthritis were less likely to access health care, use assistive devices, receive information on arthritis and its management or partake in self-management compared to those who did know their type. Based on their demographic characteristics, they may also be likely to have lower health literacy. Our findings point to the need for education not only for people with arthritis but also for primary health care providers about how to clearly communicate information about the diagnosis and management of arthritis. It also reinforces the importance of being clear that information about arthritis, such as the benefits of exercise, use of assistive devices, and pain control, applies to all people with arthritis regardless of diagnosis.

## Supporting information

S1 Table Sociodemographic and life-style variables: Canadian Community Health Survey 2008 (merged for analysis with data from the Survey of Living with Chronic Disease in Canada–Arthritis Component 2009). (DOCX)

S2 Table Arthritis-related variables: Survey of Living with Chronic Disease in Canada–Arthritis Component 2009. (DOCX)

S3 Table Healthcare utilization, medication use, assistive device use, receipt of information and self-management variables: Survey of Living with Chronic Disease in Canada–Arthritis Component 2009. ^a^Change in wording reflects current preferred terminology.
(DOCX)
