# Supplementary material for: I don’t know what type of arthritis I have: A population-based comparison of people with arthritis who knew their specific type and those who didn’t
Source: PLoS One. 2022 Jun 21;17(6):e0270029. doi: 10.1371/journal.pone.0270029 (PMC9212124; doi:10.1371/journal.pone.0270029)
Supplement: S1 Table — (DOCX) [file pone.0270029.s001.docx]

**Table S1: Sociodemographic and life-style variables: Canadian Community Health Survey 2008 (merged for analysis with data from the Survey of Living with Chronic Disease in Canada – Arthritis Component 2009)**

| **Characteristic** | **Survey question(s)** | **Response options** | **Analyzed groups** |
| --- | --- | --- | --- |
| Age | What is your age? | Range: 0-130 | 1. 20-44 years 2. 45-54 years 3. 55-64 years 4. 65-74 years 5. 75+ years |
| Sex | Is the respondent male or female? | 1. Male 2. Female | Same as response options |
| Education | Statistics Canada derived variable | 1. Less than secondary school graduation 2. Secondary school graduation 3. Some post-secondary 4. Post-secondary graduation | 1. Secondary school graduation or less 2. At least some post-secondary |
| Low household Income | Statistics Canada derived variable | Deciles 1-10 | 1. Low income (Deciles 1-3) 2. Not low income (Deciles 4-10) |
| Marital status | What is your marital status? | 1. Married 2. Common-law 3. Widowed 4. Separated 5. Divorced 6. Single, never married | 1. Married/Common-law 2. Widowed/Separated/Divorced 3. Single, never married |
| Cultural background | Statistics Canada derived variable | 1. White 2. Black 3. Korean 4. Filipino 5. Japanese 6. Chinese 7. South Asian 8. Southeast Asian 9. Arab 10. West Asian 11. Latin American 12. Other racial or cultural origin 13. Multiple racial/cultural origins 14. Aboriginal (N.A., Indian, Metis, Inuit) | 1. White 2. Aboriginal 3. Other |
| Area of residence | Statistics Canada derived variable | 1. Rural 2. Urban | Same as response options |
| BMI | Statistics Canada derived variable | Calculated from self-reported height (m) and weight (kg): kg/m^2^  Range: 8.34 - 135.31 | 1. Underweight/Normal weight 2. Overweight 3. Obese |
| Smoking status | Statistics Canada derived variable | 1. Daily smoker 2. Occasional smoker (Former daily smoker) 3. Always an occasional smoker 4. Former daily smoker 5. Former occasional smoker 6. Never smoked | 1. Current/Former smoker 2. Never smoked |
| Level of physical activity | Statistics Canada derived variable | 1. Active 2. Moderately active 3. Inactive | 1. Active 2. Inactive |
| Alcohol consumption | Statistics Canada derived variable | 1. Regular drinker 2. Occasional drinker 3. Did not drink in the last 12 months | 1. Regular drinker 2. Non-regular drinker |
