# Supplementary material for: I don’t know what type of arthritis I have: A population-based comparison of people with arthritis who knew their specific type and those who didn’t
Source: PLoS One. 2022 Jun 21;17(6):e0270029. doi: 10.1371/journal.pone.0270029 (PMC9212124; doi:10.1371/journal.pone.0270029)
Supplement: S2 Table — (DOCX) [file pone.0270029.s002.docx]

**S2 Table. Arthritis-related variables: Survey of Living with Chronic Disease in Canada – Arthritis Component 2009.**

| **Characteristic** | **Survey question(s)** | **Response options** | **Analyzed groups** |
| --- | --- | --- | --- |
| Duration of arthritis | Statistics Canada derived variable | Calculated from self-reported age of diagnosis (age_d) and age at the time of the survey (age_c): age_c – age_d | (1) 0-5 years  (2) 6-10 years  (3) 11-19 years  (4) 20+ years |
| Number of symptomatic joint sites | In the past month, which joints have been painful? | (1) Neck  (2) Right shoulder  (3) Left shoulder  (4) Right elbow  (5) Left elbow  (6) Right wrist  (7) Left wrist  (8) Right hand/fingers/thumb  (9) Left hand/fingers/thumb  (10) Back  (11) Right hip  (12) Left hip (13) Right knee  (14) Left knee  (15) Right ankle  (16) Left ankle  (17) Right foot/toes  (18) Left foot/toes  (19) Other | (1) 0 joint sites  (2) 1 joint site  (3) 2-3 joint sites  (4) 4+ joint sites |
| Joint pain | In the past month, how often have you experienced joint pain? | (1) Always  (2) Often  (3) Sometimes  (4) Rarely  (5) Never | (1) Severe (7+) and frequent (always or often) joint pain  (2) Not severe and frequent joint pain |
|  | Please tell me what number best describes, on average, how bad your joint pain  was during the past month. Answer with a number between 1 and 10; 1 means  "little pain", while 10 means "pain as bad as it could be". On average, in the past  month, how bad was your joint pain? | Integer 1-10 |  |
| Fatigue | In the past month, how often have you experienced fatigue? | (1) Always  (2) Often  (3) Sometimes  (4) Rarely  (5) Never | (1) Severe (7+) and frequent (always or often) fatigue  (2) Not severe and frequent fatigue |
|  | Please tell me what number best describes, on average, how bad your fatigue  was during the past month. Answer with a number between 1 and 10; 1 means  "little fatigue", while 10 means "fatigue as bad as it could be". On average, in the past  month, how bad was your fatigue? | Integer 1-10 |  |
| Sleep | In the past month, how much did your arthritis limit you in getting a good night’s sleep? | (1) A lot  (2) A little  (3) Not at all | (1) Affected sleep a lot  (2) Did not affect sleep a lot |
| Activities | In the past month, how much did your arthritis limit you  …in bathing or dressing yourself?  …in getting around the house?  …in doing household chores?  …in running errands or shopping?  …in activities such as recreation, leisure, hobbies, or social activities? | (1) A lot  (2) A little  (3) Not at all | (1) ≥3 activities limited a lot  (2) <3 activities limited a lot |
| Self-rated general health | In general would you say your health is excellent, very good, good, fair, or poor? | (1) Excellent  (2) Very good  (3) Good  (4) Fair  (5) Poor | (1) Fair/poor general health  (2) Good or better general health |
| Self-rated mental health | In general would you say your mental health is excellent, very good, good, fair, or poor? | (1) Excellent  (2) Very good  (3) Good  (4) Fair  (5) Poor | (1) Fair/poor mental health  (2) Good or better mental health |
| Overall impact of arthritis on life | Overall, how much does your arthritis affect your life? | (1) Not at all  (2) A little bit  (3) Moderately  (4) Quite a bit  (5) Extremely | (1) Quite a bit/extremely impacted  (2) Moderately impacted or less |
| Life stress | Thinking about the amount of stress in your life, would you say that most days are not at all stressful, not very stressful, a bit stressful, quite a bit stressful, extremely stressful? | (1) Not at all stressful  (2) Not very stressful  (3) A bit stressful  (4) Quite a bit stressful  (5) Extremely stressful | (1) Quite a bit/extremely stressful  (2) Moderately stressful or less |
