# Supplementary material for: I don’t know what type of arthritis I have: A population-based comparison of people with arthritis who knew their specific type and those who didn’t
Source: PLoS One. 2022 Jun 21;17(6):e0270029. doi: 10.1371/journal.pone.0270029 (PMC9212124; doi:10.1371/journal.pone.0270029)
Supplement: S3 Table — aChange in wording reflects current preferred terminology. (DOCX) [file pone.0270029.s003.docx]

**S3 Table. Healthcare utilization, medication use, assistive device use, receipt of information and self-management variables: Survey of Living with Chronic Disease in Canada – Arthritis Component 2009.**

| **Characteristic** | **Survey question(s)** | **Response options** | **Analyzed groups** |
| --- | --- | --- | --- |
| **Healthcare utilization** |  |  |  |
| Primary care physician^a^ | In the past 12 months, have you seen, or talked to a family doctor or general practitioner about your arthritis? | (1) Yes  (2) No | Same as response options |
| Orthopaedic surgeon | In the past 12 months, have you seen, or talked to an orthopaedic surgeon about your arthritis? | (1) Yes  (2) No | Same as response options |
| Rheumatologist | In the past 12 months, have you seen, or talked to a rheumatologist about your arthritis? | (1) Yes  (2) No | Same as response options |
| Internist | In the past 12 months, have you seen, or talked to a general internist surgeon about your arthritis? | (1) Yes  (2) No | Same as response options |
| Physio or occupational therapist | In the past 12 months, have you seen, or talked to a physiotherapist or occupational therapist about your arthritis? | (1) Yes  (2) No | Same as response options |
| Pharmacist | In the past 12 months, have you seen, or talked to a pharmacist about your arthritis? | (1) Yes  (2) No | Same as response options |
| Complementary practitioners | In the past 12 months, have you seen, or talked to a complementary or alternative health care practitioner such as a massage therapist or an osteopath about your arthritis? | (1) Yes  (2) No | Same as response options |
| **Medication use** |  |  |  |
| Prescription | In the past month, did you take prescription medications for your arthritis? | (1) Yes  (2) No | Same as response options |
| Non-prescription | In the past month, did you take non-prescription medications (that is over-the-counter products) such as pills, rubs or creams, excluding natural health products, for your arthritis? | (1) Yes  (2) No | Same as response options |
| Natural product | In the past month, did you take natural health products, that is vitamin, mineral or herbal supplements or other  natural treatments for your arthritis? | (1) Yes  (2) No | Same as response options |
| **Assistive device use** |  |  |  |
| Currently use | Statistics Canada derived variable | (1) Yes  (2) No | Same as response options |
| **Information received** |  |  |  |
| Type of arthritis | Have you ever received information on the type of arthritis you have? | (1) Yes  (2) No | Same as response options |
| Arthritis management | Have you ever received information on  …how to protect your joints, for example, by using orthotics such as splints or  braces or by using other assistive devices?  …energy conservation techniques, for example, how to manage fatigue by  balancing activities and rest, or by developing good sleep habits?  …the correct use of prescription arthritis medication?  …where to find additional information to help you manage your arthritis? | (1) Yes  (2) No | Same as response options |
| Emotional impact | Have you ever received information on the emotional impact of having arthritis, for example, how to deal with your emotions or stress? | (1) Yes  (2) No | Same as response options |
| Where to find arthritis support info | Have you ever received information on where to receive support to help you cope with your arthritis, for example, support groups or self-management programs? | (1) Yes  (2) No | Same as response options |
| Feel has enough arthritis info | Overall, do you feel that you have enough information to help you manage your  arthritis? | (1) Yes  (2) No | Same as response options |
| **Arthritis self-management** |  |  |  |
| Taken class | Have you ever taken a course or class to teach you how to manage problems related to your arthritis? | (1) Yes  (2) No | Same as response options |
| Exercise | Do you currently exercise or participate in physical activities to help manage problems related to your arthritis? | (1) Yes  (2) No | Same as response options |
| Lose/control weight | Are you currently trying to control your weight or lose weight to help manage your arthritis? | (1) Yes  (2) No | Same as response options |
| Use facilities/services/programs | In the past 12 months, have you used any community-based facilities, services or  programs such as fitness facilities, the Arthritis Society self-management program,  or telephone information or support lines, to help manage your arthritis? | (1) Yes  (2) No | Same as response options |

^a^Change in wording reflects current preferred terminology.
